# Retraction: Unlocking the power of L1 regularization: A novel approach to taming overfitting in CNN for image classification

**DOI:** 10.1371/journal.pone.0351271

**Published:** 2026-06-23

**Authors:** The PLOS One Editors

The *PLOS One* Editors retract this article [1] due to concerns about potential manipulation of the publication process. These concerns call into question the validity and provenance of the reported results. We regret that the issues were not identified prior to the article’s publication.

RS, FW, SA, YM, JK, and YL did not agree with the retraction. AA either did not respond directly or could not be reached.
